# Isolation and Identification of a Recombinant Porcine Epidemic Diarrhea Virus With a Novel Insertion in S1 Domain

**DOI:** 10.3389/fmicb.2021.667084

**Published:** 2021-04-20

**Authors:** Dongliang Li, Yongtao Li, Yunchao Liu, Yumei Chen, Wenqiang Jiao, Hua Feng, Qiang Wei, Jucai Wang, Yuhang Zhang, Gaiping Zhang

**Affiliations:** ^1^College of Veterinary Medicine, Henan Agricultural University, Zhengzhou, China; ^2^Henan Provincial Key Laboratory of Animal immunology, Henan Academy of Agricultural Sciences, Zhengzhou, China; ^3^School of Life Sciences, Zhengzhou University, Zhengzhou, China

**Keywords:** porcine epidemic diarrhea virus, spike protein, novel insertion, recombination, pathogenicity

## Abstract

Porcine epidemic diarrhea virus (PEDV) is the major pathogen that causes diarrhea and high mortality in newborn piglets with devastating impact to the pig industry. Recombination and mutation are the main driving forces of viral evolution and genetic diversity of PEDV. In 2016, an outbreak of diarrhea in piglets occurred in an intensive pig farm in Central China. A novel PEDV isolate (called HNAY) was successfully isolated from clinical samples. Sequence analysis and alignment showed that HNAY possessed 21-nucleotide (nt) insertion in its S1 gene, which has never been reported in other PEDV isolates. Moreover, the sequence of the insertion was identical with the sequence fragment in PEDV N gene. Notably, the HNAY strain exhibited two unique mutations (T500A and L521Y) in the neutralizing epitopes of the S1 protein that were different from those of other PEDV variant strains and CV777-based vaccine strains. Additionally, PEDV HNAY might be derived from a natural recombination between two Chinese variant PEDV strains. Animal experiments demonstrated that HNAY displayed higher pathogenicity compared with two other clinical isolates. This study lays the foundation for better understanding of the genetic evolution and molecular pathogenesis of PEDV.

## Introduction

Coronaviruses (CoVs) belong to RNA viruses and infect different vertebrates, such as mammalian and avian species (Durham et al., 1979; Law et al., 2005). As an important member of CoVs, porcine epidemic diarrhea virus (PEDV) can cause an acute enteritis disease, which is characterized by vomiting, diarrhea and dehydration, leading to high mortality in newborn piglets (Song and Park, 2012). The genome of PEDV is about 28 kb in size and composed of at least six open reading frames (ORFs) including ORF1a, ORF1b, spike (S), envelope (E), membrane (M), and nucleocapsid (N) (Wang et al., 2019). Among them, S protein is one of the most important structural protein due to its involvement in multiple functions, such as virus attachment, receptor binding, virus-cell membrane fusion, and inducing the production of virus neutralizing antibodies in the host (Vaughn et al., 1995; Vennema et al., 1998; Vijgen et al., 2005; Hasoksuz et al., 2007; Lorusso et al., 2008). Therefore, it is very necessary to continuously monitor the genetic alterations of PEDV S gene in the field (Park et al., 2014; Zhao et al., 2017).

Since its first appearance in Europe in 1971, PEDV has caused considerable economic losses, especially in Asia (Pensaert and de Bouck, 1978; Sun et al., 2016). Since 2010, PEDV variant strain has emerged in Asia, which can infect the pigs of all ages but most severely in suckling pigs, reaching up to 95% mortality (Bi et al., 2012; Li et al., 2012; Pan et al., 2012). In 2013, the first outbreak of PEDV in the United States (US) was reported, and then spread to Canada and Mexico (Stevenson et al., 2013; Chen et al., 2014; Pasick et al., 2014). Phylogenetic analyses showed the emergent US PEDV strains are most closely related to a Chinese strain detected in 2012 (Huang et al., 2013). From a broad perspective, phylogenetic analysis based on S gene can divide PEDVs into two major genotypes in the US: the original non-S-INDEL (S-INDEL standing for insertions/deletions in the S gene) and the variant S-INDEL (Vlasova et al., 2014; Lin et al., 2016). PEDV strains which were first identified in the US in 2013 with high virulence are classified as the non-S INDEL strains (Stevenson et al., 2013). The new variant strains which were reported in the US in 2014 with relatively milder virulence are termed as S-INDEL strains (Wang et al., 2014). In addition, PEDVs can be divided into genome 1 (G1) and genome 2 (G2) clades and further divided into four subgroups: G1a, G1b, G2a, and G2b (Wang et al., 2016). Despite they have relatively conserved neutralizing epitopes, commercial vaccines based on CV777 strains cannot provide complete protection in piglets against variant PEDV infections which are currently circulating in China and the US (Zhu et al., 2019). Monitoring the genetic alterations in the S gene of PEDV could provide more clues for understanding the epidemic characteristics of PEDV and adjusting the immunization program of PED in pig farms.

Henan province, located in the center of China, is the largest pig-producing province in China. Since 2010, severe diarrhea outbreaks caused by variant PEDV frequently occurs in this area. In our routine epidemiological investigation, 52 sequences of the full-length S genes and 9 complete genomes of PEDV have been determined, indicating the complexity of PEDV in Henan (Su et al., 2016). In this study, a PEDV strain with novel insertion in its S gene (called HNAY) was successfully isolated from an intensive farm in Henan in 2016. We determined the genetic relationships, variant characteristics, potential recombination features, and the clinical characterization of HNAY. Our findings could provide valuable information for PED outbreaks in Central china and contribute to prevent and control of PED.

## Materials and Methods

### Sample Collection, Reverse Transcription (RT)-PCR, and Sequencing

Intestine and fecal samples were collected from pig farms in Henan, China during 2016–2018 and stored at –80°C. These samples came from piglets with clinical signs including vomiting, and severe diarrhea. Total RNA was extracted from the small intestine samples using TaKaRa MiniBEST Viral RNA/DNA Extraction Kit Ver.5.0 (TaKaRa, Dalian, China). Viral cDNA was generated via reverse transcription using PrimeScript reverse transcriptase (TaKaRa, Dalian, China) according to the manufacturer’s instructions. The samples were examined for PEDV by RT-PCR (Li et al., 2018). Three samples tested PEDV positive were sequenced for complete genome as described in previous study (Wang et al., 2013). Purified PCR products were cloned into PMD-19T vector and recombinant DNA clones were sequenced by the BigDye Terminator v3.1 Cycle Sequencing kit (Biotech Company, China).

### Phylogenetic and Recombination Analysis

Complete genome sequence of PEDV and their S gene were aligned using ClustalW in DNASTAR7.1. Phylogenetic tree of PEDV was constructed using neighbor-joining (NJ) method with 1,000 bootstrap replicates in MEGA7. The sequences of reference strains are downloaded from GenBank database (Table 1). Potential recombination in the genome of PEDV HNAY were assessed by the Recombination Detection Program v4 (RDP4) which was supported by ≥6 programs.

**TABLE 1:** PEDV strains used in this study.

Strain name	Countries	Time	Genotype	Accession no.	Strain name	Countries	Time	Genotype	Accession no.
AH2012	China	2012	G2a	KC210145	CHSD2014	China	2014	G2b	KX791060.1
CH ZMDZY 11	China	2011	G2a	KC196276.1	AH2012-12	China	2012	G2b	KU64683.1
Kansas125	USA	2014	G2a	KJ645701.1	ZL29	China	2015	S-INDEL	KU847996
KNU-1305	Korae	2013	G2a	KJ662670.1	KNU-1406-1	Korea	2014	S-INDEL	KM403155.1
KGS-1	Japan	2013	G2a	LC063814.1	KCH-2	Japan	2014	S-INDEL	LC063845.1
NIG-1	Japan	2014	G2a	LC063830.1	Indiana12.83	USA	2013	S-INDEL	KJ645635.1
MN	USA	2013	G2a	KF468752.1	Iow106	USA	2013	S-INDEL	KJ645695.1
MEX124	MEX	2014	G2a	KR206669	OH851	USA	2013	S-INDEL	KJ399978.1
EHM-1	Japan	2014	G2a	LC063812	Ohio126	USA	2014	S-INDEL	KJ645702.1
PEDV-Hjms	China	2016	G2a	KY007139.1	MYZ1	USA	2013	S-INDEL	LC063846
TC PC177-P2	USA	2013	G2a	KM392229.1	aDR13	Korea	2003	G1	JQ023162.1
TTR-2	Japan	2014	G2a	LC063828.1	CV777	Belgium	1978	G1	AF353511.1
CH JX-2013	China	2015	G2a	KJ526096.1	JS2008	China	2008	G1	KC109141.1
GDS28	China	None	G2a	MH726372.1	SM98	Korea	1998	G1	GU937797.1
PC22A	USA	2013	G2a	KY499262.1	vDR13	Korea	1999	G1	JQ023161.1
Colorado	USA	2013	G2a	KF272920.1	SD-M	China	2012	G1	JX560761
HNAY 2015	China	2015	G2a	KR809885.1	LZC	China	2006	G1	EF185992
HNZZ47	China	2016	G2a	KX981440.1	CHM	China	2013	G1	KM88144
Missouri270	USA	2015	G2a	KR265846.1	KPEDV9	Korea	2013	G1	KF898124.1
LC	China	2012	G2b	JX489155.1	HNXX^*a*^	China	2016	G2a	MT338517.1
AJ1102	China	2011	G2b	JX188454.1	HNAY^*a*^	China	2016	G2	MT338518.1
CH SCZG	China	2017	G2b	MH061337	HB^*a*^	China	2016	G2b	KY928065.1

*^*a*^The PEDV strains isolated in current study.*

### Protein Structure Prediction

According to the crystal structure of S protein of PEDV USA/Colorado/2013 available in the PDB database (accession code 6VV5) as the input model, the three-dimensional structure of the S protein of HNAY was generated via SWISS-MODEL Homology Modeling server and figures were made with PymoL (Version 1.0 Schrödinger, LLC; Wrapp and McLellan, 2019).

### Virus Isolation and Immunofluorescence Assay

Vero cells were used for viral isolation as previously described (Oka et al., 2014). Cells were inoculated with sterile supernatants of prepared samples, maintained in Dulbecco’s modified Eagle’s medium (DMEM; HyClone) with 10 μg/mL trypsin (Life Technologies) and monitored daily for cytopathic effect (CPE). All the PEDV isolates were passed in Vero cells for 10 generations, and the 10th-generation of plaque purified viruses were chosen for the following experiments.Then, the cells were fixed with anhydrous ethanol when 70% of cells showed CPE. Finally, an immunofluorescence assay (IFA) was performed with a mouse anti-N protein monoclonal antibody diluted 1:1,000, and with a fluorescence isothiocyanate (FITC)-conjugated goat anti-mouse secondary antibody (1:200 dilution), 4’,6-diamidino-2-phenylindole (DAPI) was used for nucleic acids staining. The plates were examined using a fluorescence microscope.

### Determination for the Viral Growth Curve

To determine the growth curve of PEDV strains of HNAY, HB, and HNXX, viruses were inoculated onto cell monolayers at MOI of 0.1. After adsorption at 37°C for 1 h, the cells were maintained with DMEM containing 10 μg/mL trypsin at 37°C with 5% CO_2_. Cells were collected for virus titration every 12 h post infection. After centrifugation, the supernatants were collected and the median tissue culture infective dose per milliliter (TCID_50_/mL) was determined with a microtitration infection assay described by Reed and Muench (1938). Virus titration at different time points was performed in triplicates.

### Piglet Challenge Experiment

To determine the virulence of PEDV isolates, 42 two-day-old piglets, which were confirmed negative for PEDV and other common viral pathogens in pigs by PCR or RT-PCR, were chosen for challenge experiment. Then they were randomized into four groups by weight with 10 pigs in each group, and housed in separate rooms. The infection groups were orally challenged with 0.5 mL of the 10th passage of plaque purified PEDV HNAY, HB, and HNXX, respectively, at 10^5^ TCID_50_/mL. The 10 piglets of control group were inoculated with equal volume of medium. All the animals were monitored daily for clinical signs. Rectal swabs were collected for scoring fecal denseness according to previous studies (Lin et al., 2015). The fecal viral RNA loads were quantified by real-time RT-qPCR (Su et al., 2018). The animal experiment was approved by Institutional Animal Care and Use Committee of Henan Academy of Agriculture Science (LLSC100085).

### Histopathological Examination and Immunohistochemistry

Intestine and other major organs were collected and fixed for pathological examination. Samples of duodenum, jejunum, ileum, caecum, colon, rectum, and mesenteric lymph nodes were collected, and fixed in formalin. After 48 h, the jejunum was stained with haematoxylin and eosin (H&E) and the villous height-to-crypt depth (VH:CD) ratios were calculated according to previous study (Jung et al., 2014). Serial sections of ileums were evaluated for viral antigen by immunohistochemistry (IHC) using a mouse anti-N protein monoclonal antibody and biotinylated goat anti-mouse antibody as the secondary antibody with 3,3′-diaminobenzidine (DAB) as the chromogen (Lin et al., 2011).

### Statistical Analysis

All data are expressed as means ± standard deviations (SD) and were analyzed with the GraphPad Prism software (GraphPad Software Inc.). Statistical analysis was performed using one-way ANOVA or two-way ANOVA followed by *t*-test. Differences were considered statistically significant at *P* < 0.05.

## Results

### Viral Isolation and Identification

**FIGURE 1:**
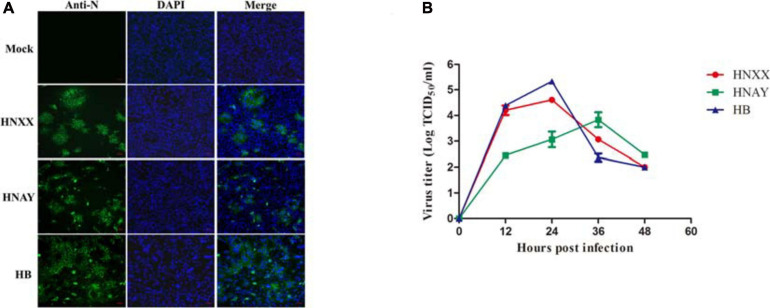
PEDV isolates replicated in Vero cells. **(A)** Detection of PEDV infection in Vero cells by IFA. **(B)** Growth curves of three PEDV isolates at passage 5 in Vero cells. Data is presented as mean ± SD by triplicates.

To determine causative agent associated with diarrhea in piglets, total RNA of clinical samples were extracted and subjected to RT-PCR. PEDV positive samples were processed and used for virus isolation on Vero cell as described previously (Chen et al., 2014). CPEs were observed on cells inoculated with PEDV positive samples, characterized by the rounding up, enlarging and detachment of cells as well as the formation of syncytia. In the end, three PEDV strains were successfully isolated, named HNAY, HNXX, and HB, respectively. The IFA results showed that PEDV specific fluorescence was observed in the inoculated cells, but not in the non-infected cells (Figure 1A). The viral titres of three PEDV isolates were determined in Vero cells. The titers of HNXX and HB reached the maximum (10^4.6^ TCID_50_/mL and 10^5.3^ TCID_50_/mL) at 24 hpi, whereas the peak virus titer of HNAY only reached 10^3.8^TCID_50_/mL at 36 hpi (Figure 1B).

### Sequence Analysis of the PEDVs

The genome of the isolates were sequenced and submitted to GenBank (accession numbers MT338517.1, MT338518.1, and KY928065.1). From nt identity analysis of the PEDV genome, HNAY exhibited 98.0–98.5% identity with the reference S-INDEL strains, 97.9–98.9% identity with the reference G2a strains, 98.0–98.5% identity with the reference G2b strains, and 96.4–97.6% identity with the reference G1 strains. HNAY shared 97.9 and 97.6% identity with HB (G2b), and HNXX (G2a), respectively (Table 2). From the nt identity analysis of PEDV S genes, HNAY exhibited 94–96.9% identity with the S-INDEL strains, 98–99.1% identity with the G2a strains, 97.5–98.5% identity with the G2b strains, and 93.7–94.3% identity with the G1 strains. However, HNAY shared 98.5 and 98.6% with HNXX, and HB, respectively (Table 3). From the amino acid (aa) identity analysis of PEDV S proteins, HNAY exhibited 92.9–96.2% identity with the S-INDEL strains, 98.4–99% identity with the G2a strains, 97.7–98.1% identity with the G2b strains, and 92.6–94.4% identity with the G1 strains. However, HNAY shared 97.7 and 98.1% with HNXX, and HB, respectively (Table 4).

**TABLE 2:** Nucleotide sequence identity (%) of complete genome of different PEDV strains.

Strain name	HNXX	HNAY	HB	Genotype	Strain name	HNXX	HNAY	HB	Genotype
AH2012	98.6	98.8	98.0	G2a	AJ1102	98.2	98.4	98.2	G2b
CH ZMDZY11	98.8	98.8	97.9	G2a	LC	98.2	98.4	98.2	G2b
Kanasas125	98.5	98.6	97.8	G2a	ZL29	98.2	98.1	97.2	S-INDEL
KNU-1305	98.6	98.8	98.0	G2a	ohio126	98.1	98.3	97.4	S-INDEL
KGS-1	98.2	98.4	97.5	G2a	Indiana12.83	98.3	98.3	97.4	S-INDEL
NIG-1	98.2	98.5	97.5	G2a	Iow106	98.2	98.2	97.4	S-INDEL
MN	98.6	98.8	98.0	G2a	KNU-01406-1	98.3	98.2	97.5	S-INDEL
MEX124	98.5	98.6	97.8	G2a	KCH-2	97.8	98.1	97.1	S-INDEL
EHM-1	98.2	98.4	97.5	G2a	OH851	98.4	98.3	97.5	S-INDEL
PEDV-Hjms	98.3	98.4	97.7	G2a	MYZ1	98.7	98.4	97.7	S-INDEL
TC PC177-P2	98.6	98.9	98.0	G2a	CV777	96.4	96.5	96.4	G1
TTR-2	98.5	98.9	97.9	G2a	LZC	96.1	96.2	96.1	G1
CH JX-2013	98.5	98.8	98.0	G2a	VSM98	96.0	96.0	96.0	G1
CH SCZG	98.4	98.7	98.2	G2a	CHM	96.1	96.1	96.0	G1
HNZZ47	98.9	99.2	98.0	G2a	JS2008	96.6	96.7	96.7	G1
HNAY 2015	98.7	99.4	98.0	G2a	SD-M	96.5	96.6	96.6	G1
GDS28	98.7	99.1	98.1	G2a	vDR13	97.2	97.4	97.1	G1
PC22A	98.9	98.7	98.0	G2a	aDR13	96.5	96.5	96.6	G1
Colorado	98.8	98.6	98.0	G2a	HNXX	100	98.5	97.6	G2a
Missouri270	98.8	98.6	97.9	G2a	HNAY	98.5	100	97.9	G2
AN2012-12	97.7	97.9	98.2	G2b	HB	97.6	97.9	100	G2b
CHSD2014	98.3	98.5	98.2	G2b					

**TABLE 3:** Nucleotide sequence identity (%) of S genes of different PEDV strains.

Strain name	HNXX	HNAY	HB	Genotype	Strain name	HNXX	HNAY	HB	Genotype
KGS-1	98.8	98.9	98.1	G2a	CH SCZG	97.9	98.5	98.3	G2b
KNU-1305	98.8	98.9	98.1	G2a	AH2012-12	97.0	97.5	98.3	G2b
MIN	98.8	98.9	98.1	G2a	ZL29	96.6	96.9	95.5	S-INDEL
NIG-1	98.8	99.0	98.1	G2a	KNU-1406-1	96.2	96.7	95.8	S-INDEL
Kansas 125	98.8	98.9	98.0	G2a	KCH-2	96.2	96.8	95.8	S-INDEL
CHZMDZY11	98.6	98.8	98.0	G2a	OH851	96.2	96.8	95.8	S-INDEL
AH2012	98.3	98.5	97.7	G2a	Ohio126	96.2	96.8	95.8	S-INDEL
MEX 124	98.7	98.8	97.7	G2a	Indiana12.83	96.3	96.8	95.8	S-INDEL
EHM-1	98.8	98.9	97.8	G2a	Iowa 106	96.2	96.8	95.8	S-INDEL
PEDV-Hjms	97.9	98.4	97.5	G2a	SD-M	93.4	94.0	93.9	S-INDEL
CH JX	98.2	98.4	97.6	G2a	MYZ1	96.3	96.8	95.6	S-INDEL
TC PC177-P2	97.5	98.0	96.6	G2a	CHM	93.2	93.9	93.6	G1
TTR-2	97.8	98.2	96.8	G2a	SM98	93.2	93.8	93.8	G1
GDS28	98.5	99.3	98.1	G2a	vDR13	95.0	94.1	93.5	G1
PC22A	98.8	98.9	97.8	G2a	aDR13	93.5	94.1	94.0	G1
Colorado	98.7	98.8	97.7	G2a	CV777	93.5	94.2	94.1	G1
HNAY 2015	98.3	99.1	98.0	G2a	JS2008	93.7	94.3	94.2	G1
HNZZ47	98.5	98.7	97.5	G2a	LZC	93.0	93.7	93.4	G1
Missouri270	98.6	98.8	97.9	G2a	KPEDV9	93.3	93.9	93.6	G1
CHSD2014	97.4	97.6	98.0	G2b	HNXX	100	98.5	97.6	G2a
LC	97.3	97.7	98.1	G2b	HNAY	98.5	100	98.6	G2
AJ1102	97.3	97.6	98.1	G2b	HB	97.6	98.6	100	G2b

**TABLE 4:** Amino acid identity (%) of S proteins of different PEDV strains.

Strain name	HNXX	HNAY	HB	Genotype	Strain name	HNXX	HNAY	HB	Genotype
KGS-1	98.1	98.8	97.9	G2a	CH SCZG	97.2	98.1	98.5	G2b
KNU-1305	98.1	98.8	97.9	G2a	AH2012-12	96.8	97.7	98.5	G2b
MIN	98.1	98.8	97.9	G2a	ZL29	95.5	96.2	95.3	S-INDEL
NIG-1	98.3	99.0	98.1	G2a	KNU-1406-1	95.2	95.9	95.1	S-INDEL
Kansas 125	98.1	98.6	97.7	G2a	KCH-2	95.3	96.0	95.2	S-INDEL
CHZMDZY11	97.6	98.4	97.4	G2a	OH851	95.2	96.0	95.1	S-INDEL
AH2012	98.1	98.5	97.5	G2a	Ohio126	95.4	96.1	95.3	S-INDEL
MEX 124	98.1	98.8	97.8	G2a	Indiana12.83	95.4	96.1	95.3	S-INDEL
EHM-1	98.2	98.9	98.0	G2a	Iowa 106	95.3	96.1	95.2	S-INDEL
PEDV-Hjms	97.5	98.3	97.3	G2a	SD-M	92.3	92.9	92.8	S-INDEL
CH JX	97.8	98.3	97.4	G2a	MYZ1	95.4	96.1	95.3	S-INDEL
TC PC177-P2	98.2	99.1	97.9	G2a	CHM	91.9	92.7	92.5	G1
TTR-2	97.9	98.7	97.6	G2a	SM98	91.9	92.7	92.5	G1
GDS28	97.6	98.7	97.9	G2a	vDR13	93.6	94.4	94.4	G1
PC22A	98.1	98.8	97.9	G2a	aDR13	92.6	93.3	93.2	G1
Colorado	98.1	98.8	98.0	G2a	CV777	92.8	93.6	93.3	G1
HNAY 2015	97.7	99.0	98.1	G2a	JS2008	92.5	93.5	93.1	G1
HNZZ47	97.3	98.0	97.2	G2a	LZC	91.8	92.6	92.3	G1
Missouri270	98.1	98.8	98.0	G2a	KPEDV9	92.9	93.5	93.1	G1
CHSD2014	97.0	97.8	98.3	G2b	HNXX	100	97.7	96.9	G2a
LC	97.3	98.1	98.6	G2b	HNAY	97.7	100	98.1	G2
AJ1102	97.3	98.1	98.6	G2b	HB	96.9	98.1	100	G2b

**FIGURE 2:**
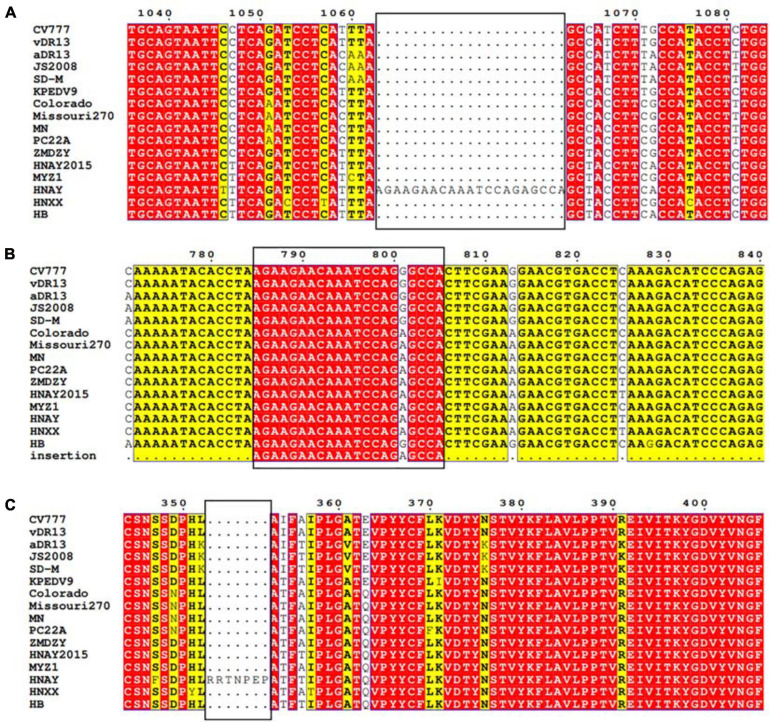
Sequence analysis of S gene of PEDV HNAY with representative PEDV strains. **(A)** Nucleotide alignment of partial S gene of PEDV HNAY with different types of reference strains. The novel insertion (AGAAGAACAAATCCAGAGCCA) in HNAY S gene is depicted as a black box. **(B)** Nucleotide alignment of partial N gene of PEDV HNAY with different types of reference strains. The black box indicates the position of 21 nucleotides inserted into the S gene in N gene. **(C)** Amino acid alignments of the partial S1 protein, insertions of seven amino acids are depicted as black boxes.

**FIGURE 3:**
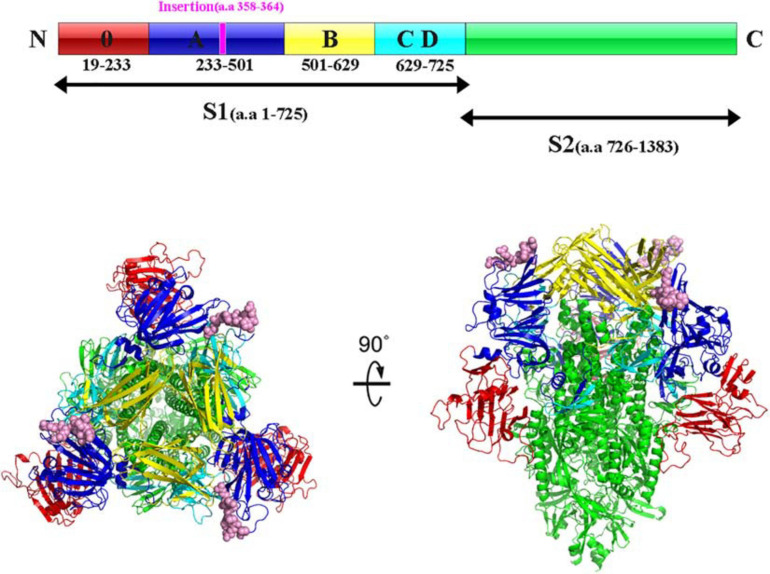
Structure prediction of S protein of PEDV HNAY in the prefusion conformation. The primary sequence of PEDV S protein can be divided into S1 and S2. The S1 region can be further divided into S10, S1A, S1B, and S1CD (in red, admiral blue, yellow, and arctic blue, respectively). The 7-aa insertion was shown in purple within S1A domain. Viewing the trimeric PEDV HNAY S protein from the membrane distal apex and a 90° view based on the crystal structure of S protein of USA/Colorado/2013. S10, S1A, S1B, S1CD, and S2 were shown in red, admiral blue, yellow, and arctic blue, respectively, and the 7-AA insertion was in pink in the crystal structure model.

Interestingly, sequence analysis showed that HNAY strain had a novel 21-nt insertion in its S1 gene at 1,063–1,083 nt (Figure 2A). Besides, the inserted sequence (AGAAGAACAAAUCCAGAGCCA) were the same as the sequence of its N gene at 785–805 nt (Figure 2B). Insertion of the deduced 7 aa was located at the 358–364 aa of PEDV S1 protein (Figure 2C). Besides, HNAY S protein structure was predicted by SWISS-MODEL according to the structure of PEDV USA/Colorado/2013 strain in PDB database (accession code 6VV5; Figure 3). Structure prediction showed that the 7-aa insertion might be located at the flexible loop (Leu354 to Ala363) at the apex of the trimer of PEDV S1A domain (Wrapp and McLellan, 2019).

**FIGURE 4:**
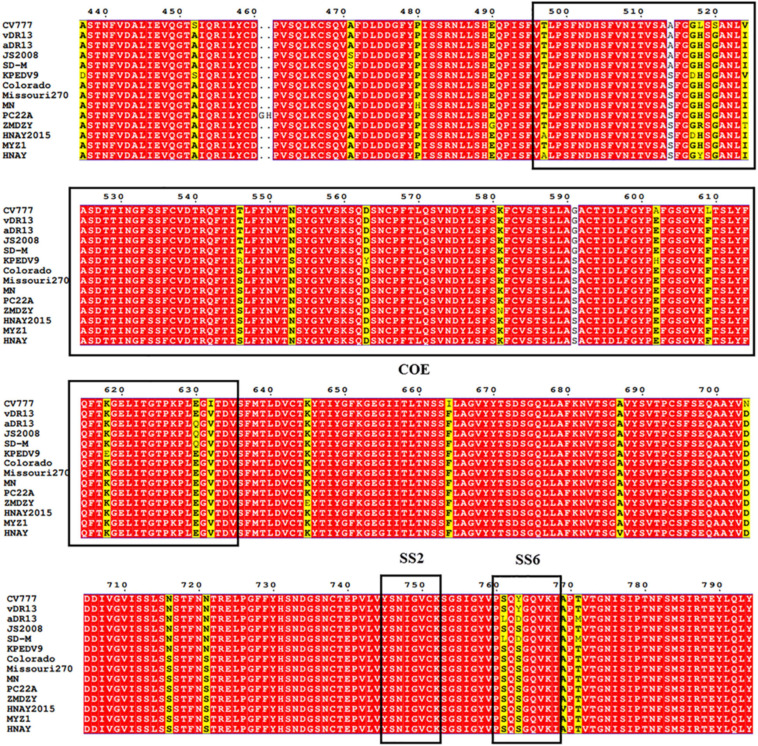
The neutralizing epitopes analysis of S protein of PEDV HNAY with reference strains. The positions of COE, SS2, and SS6 are shown in black box.

To date, three neutralizing epitopes of the PEDV S protein have been reported, including core neutralizing epitope (COE), SS2 and SS6 (Chiou et al., 2017). Multiple alignments revealed that similar to other variant strains, compared to CV777 strain, HNAY S protein possessed nine aa substitutions (A517S, S523G, V527I, T549S, G594S, A605E, L612F, I635V, and Y766S) in the COE and one amino acid substitution (Y766S) in the SS6 (Figure 4). Compared with CH/HNAY/2015 strain which was isolated in the same region as HNAY, two additional aa substitutions (D520G and H521Y) in the COE were observed in the HNAY strain. In addition to these shared mutations, HNAY strain have two unique mutations (T500A and H521Y) in the COE that were absent in other PEDV variant strains and CV777-based vaccine strains.

### Phylogenetic and Recombination Analysis

**FIGURE 5:**
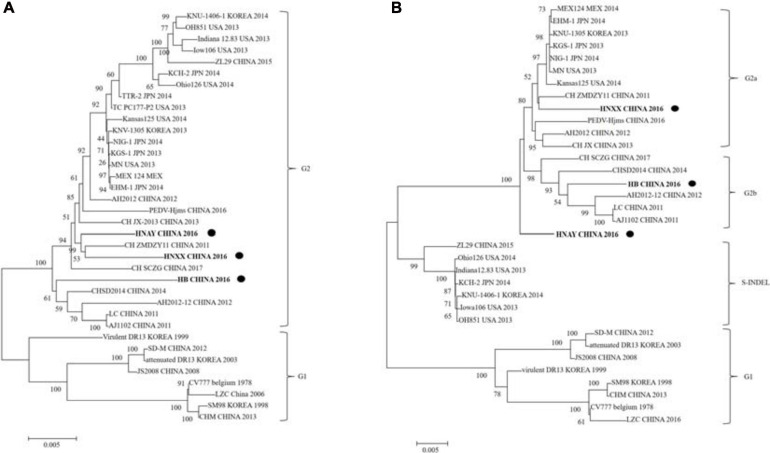
Phylogenetic analysis of PEDV complete genome and S genes. **(A)** Multiple sequence alignments were generated with Clustal X and complete genome phylogenetic tree was constructed using neighbor joining (NJ) method and supported with a bootstrap test of 1,000 replicates in MEGA7. “•” indicates the PEDV strains isolated in this study. **(B)** S gene phylogenetic tree was constructed by using the same method as above. “•” indicates the PEDV strains isolated in this study. Scale bars indicate nucleotide substitutions per site.

The phylogenetic tree based on the sequences of 3 PEDV isolates along with 33 PEDV reference strains revealed that they can be clustered into two groups, classical G1 and variant G2 (Figure 5A). G1 contains eight isolates: CV777, SM98, and DR13 isolated from Korea, and SD-M, LZC, CHM, and JS2008 from China. G2 includes many virulent strains from Japan, Korea, China, and the US. HNAY, HNXX, and HB were grouped in subgroup G2 as most of other isolates from China in the past 10 years. Furthermore, phylogenetic analysis of complete S genes delineated G2 group into 2a, 2b, and S-INDEL subgroups. HNXX and HB belongs to G2a and G2b, respectively, which was consistent with the phylogenetic analysis based on their complete genomes. Interestingly, HNAY strain belongs to a separate branch other than G2a and G2b (Figure 5B).

**FIGURE 6:**
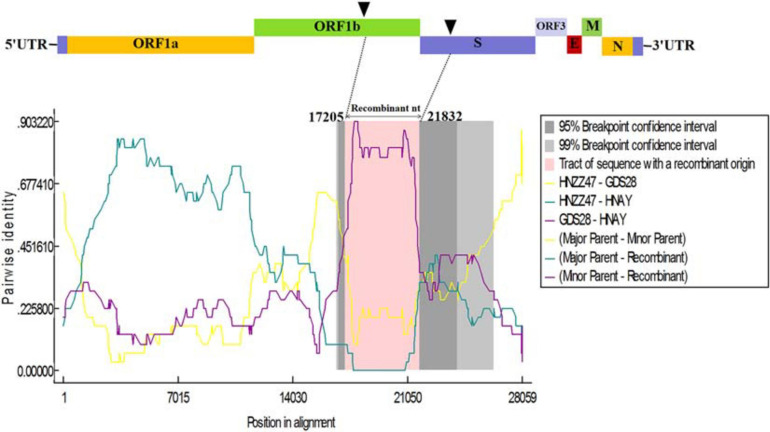
Recombination analysis of PEDV HNAY with indicated PEDV strains. The result was described using RDP method which was supported by ≥6 programs to further characterize the potential recombination events. The black arrow indicates the regions where recombination event may occur.

To further analyze the association between PEDV HNAY and reference strains, a recombination analysis was performed by using RDP4 software (Martin et al., 2015). As shown in Figure 6, PEDV HNAY might arise by the recombination of HNZZ47 and GDS28 strains, which was supported by 6 programs (Figure 6). The major parent strain of the recombination might be HNZZ47 isolated from Henan, May 2016; the minor parent strain was GDS28 which was isolated in Guangdong, December 2012. Furthermore, we also identified potential breakpoints for recombination in the ORF1b and S region (nt 17,205–21,832). This suggests that HNAY might be derived from a natural recombination between two Chinese variant PEDV strains.

### Pathogenicity of PEDV Isolates in New-Born Piglets

**FIGURE 7:**
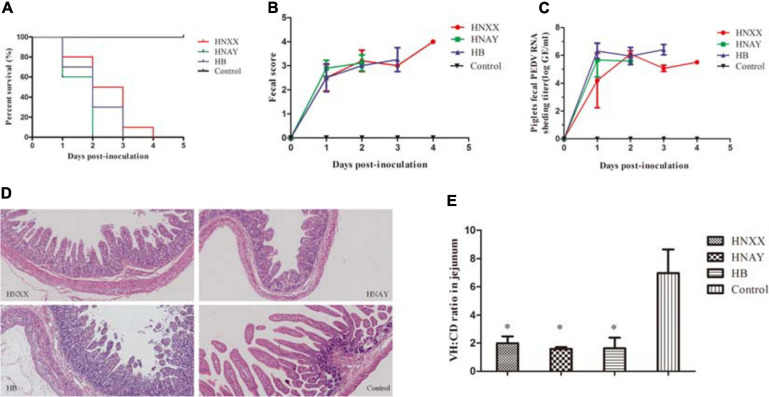
Pathogenicity analysis of PEDV isolates in new-born piglets. **(A)** Survival rate of piglets in each group. **(B)** The severity of diarrhea was scored based on clinical examination; 0 = normal and no diarrhea; 1, mild and fluidic diarrhea; 2, severe watery diarrhea; with scores of 1 or more considered diarrheic. **(C)** Fecal virus shedding in PEDV-challenged piglets by RT-qPCR. **(D)** Microscopic damage in Jejunum in piglets (Original magnifications: ×20). **(E)** Further analysis of jejunum villous high: crypt depth ratios (VH:CD).

To investigate the virulence of PEDV HNAY in piglets in comparison with that of HNXX and HB, suckling piglets were orally infected with the three PEDV strains. Clinical observation showed that piglets in challenge groups exhibited lethargy, loss of appetite, diarrhea, dehydration and vomiting at 12–20 hpi. The control pigs remained healthy. The challenged piglets began to die at 1 day post infection (dpi), and all piglets in the HNAY-, HB- and HNXX-challenged groups died within 2, 3, and 4 dpi, respectively (Figure 7A). From fecal score analysis, there was no significant difference between piglets in three groups (Figure 7B). Necropsy examinations were performed immediately after the death of the infected piglets. All the infected piglets displayed typical lesions of PED. The wall of the small intestine was thin and transparent. The small intestine and stomach were, respectively, distended and filled with curdled and undigested milk. By contrast, the intestinal organs of control piglets appeared grossly normal. Furthermore, viral RNA in fecal samples were tested positive for PEDV in challenged groups (2–6log_10_ genomic equivalents/mL). HNXX and HB reached peak at 1 dpi, but HNAY reached peak at 2 dpi (Figure 7C).

Microscopic examination of jejunum revealed moderate to severe, extensive, atrophic enteritis in three PEDV isolates infected piglets (Figure 7D). The jejunum VH:CD ratios ranged from 1.14 to 2.49 in PEDVs infected piglets. The VH:CD ratios in the three PEDVs infected piglets were lower when compared with that of non-infected pigs, but there was no obvious difference between three PEDVs infected groups (Figure 7E).

**FIGURE 8:**
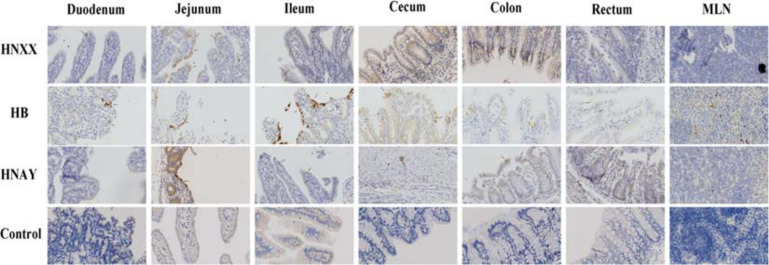
PEDV antigens detection in different intestinal tissues of piglets infected with PEDV isolates by immunohistochemistry. The representative pictures of viral antigens in duodenum, jejunum, ileum, caecum, colon, rectum, mesenteric lymph nodes of intestines from piglets of each group are shown, and antigens are depicted as arrows. Original magnifications: ×40.

In addition, PEDV antigens were detected in the duodenum, jejunum, ileum, caecum, colon, rectum and MLNs of HNXX-, HB-, and HNAY-infected piglets. IHC results showed that PEDV antigens were mainly distributed in the villus epithelial cells. Specifically, viral antigen was expressed in the colon and cecum but mainly distributed in the mucosa and submucosa of the HNXX-infected piglets; viral antigen of HB-infected piglets was expressed in the mucosal epithelium of each segment of the intestine. While the tissues from the piglets in the HNAY-infected group showed remarkable levels of viral antigen in the intestinal glands and mucosal epithelium of the jejunum, and the mucosal layer of the colon and rectum (Figure 8). The results of PEDV challenge test indicate that HNAY displayed higher pathogenicity compared with two other clinical isolates.

## Discussion

PED is one of the most critical diarrheal diseases threatening the global pig industry. In 2010, a new variant PEDV (G2 genotype) first emerged in Central China and spread rapidly throughout the country, resulting in big economic losses (Li et al., 2012). Subsequently, this type of virus spread to many countries in Asia, America and Europe (Peng et al., 2017). In 2014, another genotype of PEDV, designated as S-INDEL, was first detected in the USA (Wang et al., 2014). Since then, more and more variant PEDV strains, such as those with a large deletion in the S gene and deletions in the ORF1a gene, have been discovered worldwide (Lin et al., 2016). The S glycoprotein of coronaviruses contains two domains called S1 and S2. S1 domain contributes to receptor binding and S2 domain is thought to be associated with direct fusion between the viral and cellular membranes (Walls et al., 2016). Similar to other coronavirus S proteins, the PEDV S protein plays a critical role in viral entry and production of neutralization antibodies in the host. Most of the variant PEDV strains possessed insertion and deletions in the S0 domain (19–233 aa; Oka et al., 2014; Vlasova et al., 2014). Interestingly, HNAY was identified as a novel PEDV strain with 7-aa insertion (358–364 aa) in S1A domain (233–501 aa), and the inserted sequence was identical with the sequence of 785–805 nt in N gene. Although HNAY had higher sequence identity with G2a and G2b strains, phylogenetic analysis revealed that HNAY belonged to a single branch instead of G1, G2 or S-INDEL. In addition, a recombination event was identified in the genome of HNAY at 27,105–28,059 nt, where PEDV HNZZ47 and GDS28 were major and minor parents, respectively. Currently, none of clinically isolated PEDV strain has been reported to have multiple amino acid insertions in the S1A region. The prevalence of this type of variants in pig farms needs further epidemiological investigation.

Mutations in the S1 region of PEDV have been shown to be responsible for the alternation of viral tropism and pathogenicity in pigs (Gallagher and Buchmeier, 2001; Sato et al., 2011; Lee, 2015). In this study, HNAY-infected Vero cells were obviously characterized by cell fusion and syncytial formation, but viral titre was lower compared to that of the other two PEDV strains. Animal experiments showed that pigs that infected by HNAY showed most severe disease compared to those infected by HNXX and HB, as all of HNAY-infected piglets died within 2 dpi. Moreover, a partial of the PEDV N antigen was also found in caecum muscularis of HNAY-infected pigs, but not in other virus strains infected groups. Considering the particularity of this insertion position, further studies are required to decipher its role in receptor binding and virulence in the PEDV strain.

Recently, the atomic-resolution structure of prefusion PEDV spike protein has been resolved (Wrapp and McLellan, 2019). Similar to other class I fusion proteins in the prefusion conformation, PEDV S1 subunit is made up of a series of β-sheets, whereas the S2 subunit is almost composed of a series of discontinuous α-helices (Wrapp and McLellan, 2019). Interestingly, the 7-aa insertion happens to be in the flexible loop at the apex of the trimer (354–363 aa). However, this region was not clearly resolved in structure model of PEDV S protein (Wrapp and McLellan, 2019). Therefore, we predicted that this region may be an ideal insertion site for foreign proteins or peptides, which could provide clues for constructing recombinant PEDV strains with molecular markers in S1 domain by reverse genetics technology. Whether the insertion in this region affects the infectious characteristics and pathogenicity of PEDV needs further study.

## Conclusion

In this study, a novel 7-aa insertion in the S1 and a recombination event were detected in PEDV HNAY strain. This insertion might affect the structure of S protein, thereby directly or indirectly altering viral tropism and pathogenicity, resulting in consistent infection in pigs. Therefore, further research is needed to determine the impact of this insertion on the pathogenicity of HNAY strain. In addition, how such PEDV variants emerged and evolved in the field needs further investigations, which would significantly contribute to the prevention and control of PED outbreaks worldwide.

## Data Availability Statement

The datasets presented in this study can be found in online repositories. The names of the repository/repositories and accession number(s) can be found in the article/supplementary material.

## Ethics Statement

The animal study was reviewed and approved by the Animal Experiment Committee of Henan Academy of Agricultural Sciences.

## Author Contributions

DL, YL, and GZ wrote the manuscript and conceived and initiated the study. DL, YL, and YC devised the experimental methods. WJ and HF curated the data. JW and YZ performed animal experiments. DL prepared the original manuscript draft. YL and GZ reviewed the manuscript and edited it. All authors read and approved the final manuscript.

## Conflict of Interest

The authors declare that the research was conducted in the absence of any commercial or financial relationships that could be construed as a potential conflict of interest.

## References

[B1] Bi J. Zeng S. Xiao S. Chen H. Fang L. (2012). Complete genome sequence of porcine epidemic diarrhea virus strain AJ1102 isolated from a suckling piglet with acute diarrhea in China. *J. Virol.* 86 10910–10911. 10.1128/JVI.01919-12 22966198 PMC3457323

[B2] Chen Q. Li G. Stasko J. Thomas J. T. Stensland W. R. Pillatzki A. E. (2014). Isolation and characterization of porcine epidemic diarrhea viruses associated with the 2013 disease outbreak among swine in the United States. *J. Clin. Microbiol.* 52 234–243. 10.1128/JCM.02820-13 24197882 PMC3911415

[B3] Chiou H. Y. Huang Y. L. Deng M. C. Chang C. Y. Jeng C. R. Tsai P. S. (2017). Phylogenetic analysis of the spike (S) gene of the new variants of porcine epidemic diarrhoea virus in taiwan. *Transbound Emerg. Dis.* 64 157–166. 10.1111/tbed.12357 25903998

[B4] Durham P. J. Stevenson B. J. Farquharson B. C. (1979). Rotavirus and coronavirus associated diarrhoea in domestic animals. *N. Z. Vet. J.* 27 30–32. 10.1080/00480169.1979.34595 221870

[B5] Gallagher T. M. Buchmeier M. J. (2001). Coronavirus spike proteins in viral entry and pathogenesis. *Virology* 279 371–374. 10.1006/viro.2000.0757 11162792 PMC7133764

[B6] Hasoksuz M. Alekseev K. Vlasova A. Zhang X. Spiro D. Halpin R. (2007). Biologic, antigenic, and full-length genomic characterization of a bovine-like coronavirus isolated from a giraffe. *J. Virol.* 81 4981–4990. 10.1128/JVI.02361-06 17344285 PMC1900194

[B7] Huang Y. W. Dickerman A. W. Piñeyro P. Li L. Fang L. Kiehne R. (2013). Origin, evolution, and genotyping of emergent porcine epidemic diarrhea virus strains in the United States. *mBio* 4 e00737–13. 10.1128/mBio.00737-13 24129257 PMC3812708

[B8] Jung K. Wang Q. Scheuer K. A. Lu Z. Zhang Y. Saif L. J. (2014). Pathology of US *porcine epidemic diarrhea virus* strain PC21A in gnotobiotic pigs. *Emerg. Infect. Dis.* 20 662–665. 10.3201/eid2004.131685 24795932 PMC3966387

[B9] Law H. K. Cheung C. Y. Ng H. Y. Sia S. F. Chan Y. O. Luk W. (2005). Chemokine up-regulation in SARS-coronavirus-infected, monocyte-derived human dendritic cells. *Blood* 106 2366–2374. 10.1182/blood-2004-10-4166 15860669 PMC1895271

[B10] Lee C. (2015). Porcine epidemic diarrhea virus: an emerging and re-emerging epizootic swine virus. *Virol. J.* 12:193. 10.1186/s12985-015-0421-2 26689811 PMC4687282

[B11] Li D. Feng H. Liu Y. Chen Y. Wei Q. Wang J. (2018). Molecular evolution of porcine epidemic diarrhea virus and porcine deltacoronavirus strains in Central China. *Res. Vet. Sci.* 120 63–69. 10.1016/j.rvsc.2018.06.001 30265872 PMC7111851

[B12] Li W. Li H. Liu Y. Pan Y. Deng F. Song Y. (2012). New variants of porcine epidemic diarrhea virus, China, 2011. *Emerg. Infect. Dis.* 18 1350–1353. 10.3201/eid1808.120002 22840964 PMC3414035

[B13] Lin C. M. Annamalai T. Liu X. Gao X. Lu Z. El-Tholoth M. (2015). Experimental infection of a US spike-insertion deletion porcine epidemic diarrhea virus in conventional nursing piglets and cross-protection to the original US PEDV infection. *Vet. Res.* 46:134. 10.1186/s13567-015-0278-9 26589292 PMC4654902

[B14] Lin C. M. Jeng C. R. Hsiao S. H. Liu J. P. Chang C. C. Chiou M. T. (2011). Immunopathological characterization of porcine circovirus type 2 infection-associated follicular changes in inguinal lymph nodes using high-throughput tissue microarray. *Vet. Microbiol.* 149 72–84. 10.1016/j.vetmic.2010.10.018 21126833

[B15] Lin C. M. Saif L. J. Marthaler D. Marthaler D. (2016). Evolution, antigenicity and pathogenicity of global porcine epidemic diarrhea virus strains. *Virus Res.* 226 20–39. 10.1016/j.virusres.2016.05.023 27288724 PMC7111424

[B16] Lorusso A. Decaro N. Schellen P. Rottier P. J. M. Buonavoglia C. Haijema B. J. (2008). Gain, preservation, and loss of a group 1a coronavirus accessory glycoprotein. *J. Virol.* 82 10312–10317. 10.1128/JVI.01031-08 18667517 PMC2566247

[B17] Martin D. P. Murrell B. Golden M. Khoosal A. Muhire B. (2015). RDP4: Detection and analysis of recombination patterns in virus genomes. *Virus Evol.* 1:vev003. 10.1093/ve/vev003 27774277 PMC5014473

[B18] Oka T. Saif L. J. Marthaler D. Esseili M. A. Meulia T. Lin C. M. (2014). Cell culture isolation and sequence analysis of genetically diverse US porcine epidemic diarrhea virus strains including a novel strain with a large deletion in the spike gene. *Vet. Microbiol.* 173 258–269. 10.1016/j.vetmic.2014.08.012 25217400 PMC7126216

[B19] Pan Y. Saif L. J. Marthaler D. Esseili M. A. Meulia T. Lin C. M. (2012). Isolation and characterization of a variant porcine epidemic diarrhea virus in China. *Virol. J.* 9:195. 10.1186/1743-422X-9-195 22967434 PMC3487931

[B20] Park S. Kim S. Song D. Park B. (2014). Novel porcine epidemic diarrhea virus variant with large genomic deletion, South Korea. *Emerg. Infect Dis.* 20 2089–2092. 10.3201/eid2012.131642 25424875 PMC4257805

[B21] Pasick J. Berhane Y. Ojkic D. Maxie G. Embury−Hyatt C. Swekla K. (2014). Investigation into the role of potentially contaminated feed as a source of the first-detected outbreaks of porcine epidemic diarrhea in Canada. *Transbound Emerg. Dis.* 61 397–410. 10.1111/tbed.12269 25098383 PMC4282400

[B22] Peng J. Y. Jian C. Z. Chang C. Y. Chang H. W. (2017). “Porcine epidemic diarrhea,” in *Emerging and Re-Emerging Infectious Diseases of Livestock*, ed. Bayry J. (Cham: Springer), 10.1007/978-3-319-47426-7

[B23] Pensaert M. B. de Bouck P. (1978). A new coronavirus-like particle associated with diarrhea in swine. *Arch. Virol.* 58 243–247. 10.1007/BF01317606 83132 PMC7086830

[B24] Reed L. J. Muench H. (1938). A simple method of estimating fifty percent endpoints. *Am. J. Hyg.* 1938 493–497. 10.1093/oxfordjournals.aje.a118408

[B25] Sato T. Takeyama N. Katsumata A. Tuchiya K. Kodama T. Kusanagi K. (2011). Mutations in the spike gene of porcine epidemic diarrhea virus associated with growth adaptation in vitro and attenuation of virulence in vivo. *Virus Genes* 43 72–78. 10.1007/s11262-011-0617-5 21559974 PMC7088782

[B26] Song D. Park B. (2012). Porcine epidemic diarrhoea virus: a comprehensive review of molecular epidemiology, diagnosis, and vaccines. *Virus Genes* 44 167–175. 10.1007/s11262-012-0713-1 22270324 PMC7089188

[B27] Stevenson G. W. Hoang H. Schwartz K. J. Burrough E. R. Sun D. Madson D. (2013). Emergence of Porcine epidemic diarrhea virus in the United States: clinical signs, lesions, and viral genomic sequences. *J. Vet. Diagn. Invest.* 25 649–654. 10.1177/1040638713501675 23963154

[B28] Su Y. Liu Y. Chen Y. Xing G. Hao H. Wei Q. (2018). A novel duplex TaqMan probe-based real-time RT-qPCR for detecting and differentiating classical and variant porcine epidemic diarrhea viruses. *Mol. Cell Probes.* 37 6–11. 10.1016/j.mcp.2017.10.003 29104088

[B29] Su Y. Liu Y. Chen Y. Zhao B. Ji P. Xing G. (2016). Detection and phylogenetic analysis of porcine epidemic diarrhea virus in central China based on the ORF3 gene and the S1 gene. *Virol. J.* 13:192. 10.1186/s12985-016-0646-8 27887624 PMC5123408

[B30] Sun D. Wang X. Wei S. Chen J. Feng L. (2016). Epidemiology and vaccine of porcine epidemic diarrhea virus in China: a mini-review. *J. Vet. Med. Sci.* 78 355–363. 10.1292/jvms.15-0446 26537549 PMC4829501

[B31] Vaughn E. M. Halbur P. G. Paul P. S. (1995). Sequence comparison of porcine respiratory coronavirus isolates reveals heterogeneity in the S, 3, and 3-1 genes. *J. Virol.* 69 3176–3184. 10.1128/JVI.69.5.3176-3184.1995 7707547 PMC189021

[B32] Vennema H. Poland A. Foley J. Pedersen N. C. (1998). Feline infectious peritonitis viruses arise by mutation from endemic feline enteric coronaviruses. *Virology* 243 150–157. 10.1006/viro.1998.9045 9527924 PMC7131759

[B33] Vijgen L. Keyaerts E. Moës E. Thoelen I. Wollants E. Lemey P. (2005). Complete genomic sequence of human coronavirus OC43: molecular clock analysis suggests a relatively recent zoonotic coronavirus transmission event. *J. Virol.* 79 1595–1604. 10.1128/JVI.79.3.1595-1604.2005 15650185 PMC544107

[B34] Vlasova A. N. Marthaler D. Wang Q. Culhane M. R. Rossow K. Rovira A. (2014). Distinct characteristics and complex evolution of PEDV strains, North America, May 2013-February 2014. *Emerg. Infect. Dis.* 20 1620–1628. 10.3201/eid2010.140491 25279722 PMC4193278

[B35] Walls A. C. Tortorici M. A. Jan Bosch B. Frenz B. Rottier P. J. M. DiMaio F. (2016). Cryo-electron microscopy structure of a coronavirus spike glycoprotein trimer. *Nature* 531 114–117. 10.1038/nature16988 26855426 PMC5018210

[B36] Wang D. Fang L. Xiao S. (2016). Porcine epidemic diarrhea in China. *Virus Res.* 226 7–13. 10.1016/j.virusres.2016.05.026 27261169 PMC7114554

[B37] Wang L. Byrum B. Zhang Y. (2014). New variant of porcine epidemic diarrhea virus, United States, 2014. *Emerg. Infect. Dis.* 20 917–919. 10.3201/eid2005.140195 24750580 PMC4012824

[B38] Wang Q. Vlasova A. N. Kenney S. P. Saif L. J. (2019). Emerging and re-emerging coronaviruses in pigs. *Curr. Opin. Virol.* 34 39–49. 10.1016/j.coviro.2018.12.001 30654269 PMC7102852

[B39] Wang X. M. Niu B. B. Yan H. Gao D. S. Yang X. Chen L. (2013). Genetic properties of endemic Chinese porcine epidemic diarrhea virus strains isolated since 2010. *Arch. Virol.* 158 2487–2494. 10.1007/s00705-013-1767-7 23797760 PMC7087078

[B40] Wrapp D. McLellan J. S. (2019). The 3.1-angstrom cryo-electron microscopy structure of the porcine epidemic diarrhea virus spike protein in the prefusion conformation. *J. Virol.* 93:e00923-19. 10.1128/JVI.00923-19 31534041 PMC6854500

[B41] Zhao X. Li Z. Zeng X. Zhang G. Niu J. Sun B. (2017). Sequence analysis of the spike gene of *Porcine epidemic diarrhea virus* isolated from South China during 2011-2015. *J. Vet. Sci.* 18 237–243. 10.4142/jvs.2017.18.2.237 27515262 PMC5489471

[B42] Zhu T. Du S. Cao D. Pei Z. Guo Y. Shao H. (2019). Isolation and identification of a variant subtype G 2b porcine epidemic diarrhea virus and S gene sequence characteristic. *Infect. Genet. Evol.* 71 82–90. 10.1016/j.meegid.2019.03.015 30905773 PMC7106306

